# Apoptosis of CD4^+^CD25^high^ T Cells in Type 1 Diabetes May Be Partially Mediated by IL-2 Deprivation

**DOI:** 10.1371/journal.pone.0006527

**Published:** 2009-08-05

**Authors:** Parthav Jailwala, Jill Waukau, Sanja Glisic, Srikanta Jana, Sarah Ehlenbach, Martin Hessner, Ramin Alemzadeh, Shigemi Matsuyama, Purushottam Laud, Xujing Wang, Soumitra Ghosh

**Affiliations:** 1 The Max McGee National Research Center for Juvenile Diabetes and The Human and Molecular Genetics Center, Department of Pediatrics at the Medical College of Wisconsin and the Children's Research Institute of the Children's Hospital of Wisconsin, Milwaukee, Wisconsin, United States of America; 2 Children's Hospital of Wisconsin Diabetes Center, Pediatric Endocrinology and Metabolism, Medical College of Wisconsin, Milwaukee, Wisconsin, United States of America; 3 Department of Pharmacology, Case Western Reserve University, Cleveland, Ohio, United States of America; 4 Division of Biostatistics, Medical College of Wisconsin, Milwaukee, Wisconsin, United States of America; 5 Department of Physics & the Comprehensive Diabetes Center, University of Alabama at Birmingham, Birmingham, Alabama, United States of America; Duke-NUS Graduate Medical School, Singapore

## Abstract

**Background:**

Type 1 diabetes (T1D) is a T-cell mediated autoimmune disease targeting the insulin-producing pancreatic β cells. Naturally occurring FOXP3^+^CD4^+^CD25^high^ regulatory T cells (T_regs_) play an important role in dominant tolerance, suppressing autoreactive CD4^+^ effector T cell activity. Previously, in both recent-onset T1D patients and β cell antibody-positive at-risk individuals, we observed increased apoptosis and decreased function of polyclonal T_regs_ in the periphery. Our objective here was to elucidate the genes and signaling pathways triggering apoptosis in T_regs_ from T1D subjects.

**Principal Findings:**

Gene expression profiles of unstimulated T_regs_ from recent-onset T1D (n = 12) and healthy control subjects (n = 15) were generated. Statistical analysis was performed using a Bayesian approach that is highly efficient in determining differentially expressed genes with low number of replicate samples in each of the two phenotypic groups. Microarray analysis showed that several cytokine/chemokine receptor genes, HLA genes, *GIMAP* family genes and cell adhesion genes were downregulated in T_regs_ from T1D subjects, relative to control subjects. Several downstream target genes of the AKT and p53 pathways were also upregulated in T1D subjects, relative to controls. Further, expression signatures and increased apoptosis in T_regs_ from T1D subjects partially mirrored the response of healthy T_regs_ under conditions of IL-2 deprivation. CD4^+^ effector T-cells from T1D subjects showed a marked reduction in IL-2 secretion. This could indicate that prior to and during the onset of disease, T_regs_ in T1D may be caught up in a relatively deficient cytokine milieu.

**Conclusions:**

In summary, expression signatures in T_regs_ from T1D subjects reflect a cellular response that leads to increased sensitivity to apoptosis, partially due to cytokine deprivation. Further characterization of these signaling cascades should enable the detection of genes that can be targeted for restoring T_reg_ function in subjects predisposed to T1D.

## Introduction

Type 1 diabetes (T1D) results from the T-cell-mediated autoimmune destruction of the insulin-producing pancreatic islet β cells. This breakdown of immunological self-tolerance results in autoreactivity to islet self-antigens, and requires genetic susceptibility as well as environmental factors. Both the numerical and functional balance between killer (e.g., CD4^+^ and CD8^+^ effectors) and regulatory T-cells in the pancreatic infiltrate determines the extent of β cell destruction [1]. Although islet infiltrates have shown the presence of cytotoxic effector T-cells and pro-inflammatory cytokines [2], there is still a major void in our understanding of how these effector cells escape peripheral regulation.

Among the regulatory T-cells that actively suppress effector T-cells, the FOXP3^+^CD4^+^CD25^high^ T-cells (T_regs_) represent one of the best characterized sub-populations. There is accumulating evidence of a deficiency in either the frequency or function of T_regs_ in various human autoimmune diseases [3], as well as in the pathogenesis of T1D [4]–[7]. During the period right after disease onset, which lasts several months after clinical diagnosis, most T1D patients have some residual β-cell function [8], [9]. Our group is interested to study immune responses in the periphery related to β-cell destruction and progression of disease during this recent-onset period. We previously reported evidence for increased apoptosis of T_regs_ in recent-onset T1D subjects (all diagnosed within 1 year, henceforth referred to as “T1D subjects”) and in subjects *at-risk* for T1D [10]. This increase in T_reg_ apoptosis was found to correlate with a decline in suppressive potential of these cells. The fact that both hyperglycemic T1D subjects and normoglycemic *at-risk* subjects showed this phenomenon suggests that T_reg_ apoptosis is more a precursor to, rather than a consequence of diabetes. Although T_reg_ apoptosis is likely to be one of the peripheral imbalances in T1D, there is very little known about the pathways and genes that make T_regs_ sensitive to apoptosis during the period right after the onset of disease.

Several groups have studied expression profiles for various subsets of T-cells in both humans and mice, aimed at objectives ranging from differentiating regulatory T-cells from effector T-cells [11], [12] to understanding the FOXP3-dependent regulatory phenotype [13], [14], or studying how these cells respond to cytokine stimulation [15]–[17]. These studies have contributed significantly to a better understanding of the mechanisms underlying T_reg_ mediated tolerance under physiological conditions. There are also studies which have investigated T_reg_ expression under diseased conditions in mouse models for T1D [18], [19]. Recent studies have explored the expression signatures in peripheral blood mononuclear cells (PBMC) and in CD4^+^ T cells of human T1D and T2D subjects [20], [21]. However, expression profiles in unfractionated PBMC (or in the CD4^+^ T cell subset) comprising a heterogeneous cell population are difficult to correlate with expression changes that should occur specifically in apoptosis-sensitive T_regs_ at the onset of T1D.

In this study, we investigated the expression signature that shapes the transcriptional program within functionally deficient T_regs_ from recent-onset T1D. Expression profiles of unstimulated T_regs_ from T1D subjects reveal a cellular response that could make the cells sensitive to apoptosis, partially due to deprivation of cytokines. This global picture of pathway-specific expression signatures is a step further into dissecting T_reg_ dysfunction in the pathogenesis of T1D.

## Results

### T_regs_ from T1D subjects exhibit reduced suppressive capacity and increased apoptosis

**Figure 1:**
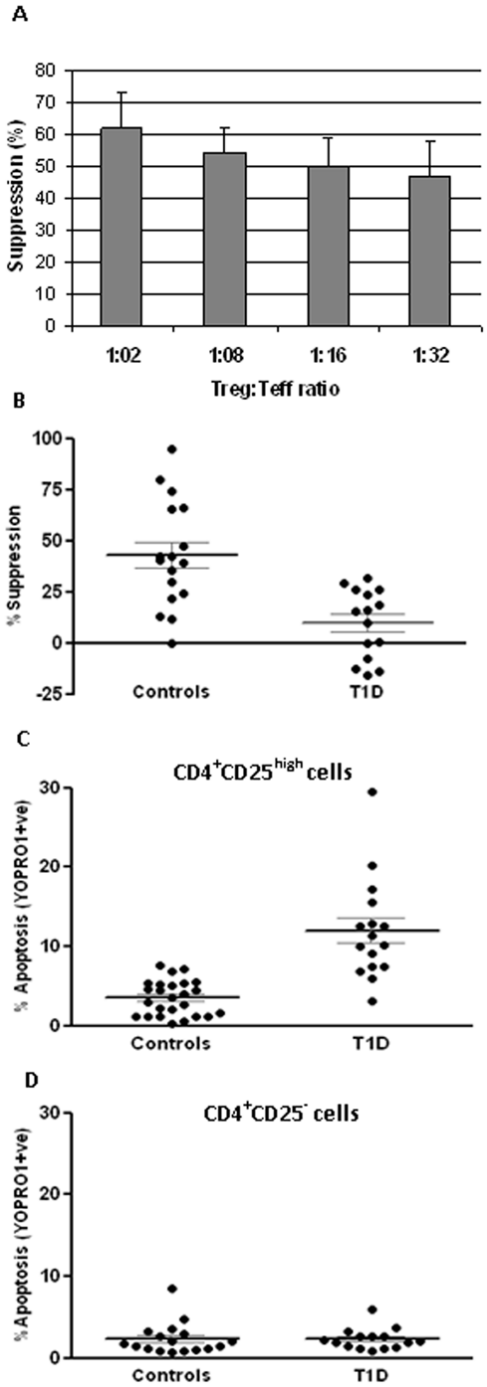
In T1D subjects, T_regs_ isolated from CD4^+^ T cells have reduced function and increased apoptosis. Standard *in vitro* suppression assay included 25,000 responder CD4^+^CD25^−^ T cells that were set up as single culture as well as with CD4^+^CD25^high^ T cells in co-culture, both with added 25,000 of irradiated PBMCs (as antigen-presenting cells) which were stimulated with aCD3-coated beads (1 µg/ml) for 5 days. Ratio of responder and CD4^+^CD25^high^ T cells was varied. Suppressors were seeded with APC in separate wells to measure their own proliferation in respond to the same stimulation. Bars are mean±SEM of three independent observations. (B) Suppression potential of isolated CD4^+^CD25^high^ T-cells measured by an *in vitro* proliferation assay in T1D (n = 17) and control (n = 15) subjects. A standardized suppression assay was performed with 2.5×10^4^ CD4^+^CD25^−^ effector T-cells (with 2.5×10^4^ irradiated PBMC -5000rad) in co-culture with CD4^+^CD25^high^ T-cells at a 1∶10 ratio (CD4^+^CD25^high^: CD4^+^CD25^−^), stimulated with aCD3 coated beads (1 ug/ml, 3 beads/per cell). Apoptosis results following YOPRO-1/7AAD staining for (C) CD4^+^CD25^high^ (T1D: n = 16, controls: n = 25) and (D) CD4^+^CD25^−^ T-cells (T1D: n = 15, controls: n = 17) are both presented as % apoptotic (YOPRO1+ve) amongst live cells (7AAD–ve).

We first investigated a possible functional deficiency in T_regs_ from T1D subjects, using a suppression assay. In a pilot suppression assay, T_regs_ from healthy control subjects were co-cultured with CD4^+^CD25^−^ effector T-cells (CD25^−^ T_effs_) at varying titrations of the T_reg_:CD25^−^ T_eff_ ratio (from 1∶2 to 1∶32) and the suppressive capacity of T_regs_ was measured using the formula described in the methods. Varying the CD25^−^ T_eff_ numbers resulted in minor changes in suppression (Figure 1A). Hence, we chose to carry out suppression assays at a 1∶10 (T_reg_: CD25^−^ T_eff_) ratio for the rest of the study. T_regs_ from T1D subjects (n = 15) showed a significant reduction in suppressive capacity compared to control subjects (n = 17) (Mann-Whitney p<0.0007) (Figure 1B). The raw CPM counts in single and co-culture were comparable within the two phenotypic groups and the low background counts confirmed that there was no media contamination (Figure S1). We further checked if ongoing spontaneous apoptosis could also be detected in T_regs_ from T1D subjects that showed reduced function. We observed that T_regs_ from T1D subjects had significantly elevated levels of apoptosis, compared to control subjects (Mann-Whitney p<0.0001) (Figure 1C), but there was no difference in apoptosis of CD25^−^ T_effs_ across the two phenotypes (Figure 1D). Also, while there was no difference in apoptosis across T_regs_ and CD25^−^ T_effs_ from the same control subjects, T_regs_ displayed higher apoptosis levels compared to CD25^−^ T_effs_ cells from the same T1D subjects. A representative flow cytometry plot for isolation of apoptotic cells is shown in Figure S2.

These results strengthened the trends reported earlier by our group in T1D subjects and *at-risk* non-diabetic individuals [10]. Furthermore, they suggest that in T1D subjects as well as in normoglycemic *at-risk* subjects, T_regs_ undergo spontaneous apoptosis in the periphery, which could result in a subsequent loss of suppressive potential. Increased apoptosis and decreased suppression of T_regs_ observed in both recent-onset T1D subjects and in at-risk individuals who have not yet been clinically diagnosed with disease, indicates that these T_reg_ defects are more a cause than an effect of the disease.

Several reports have demonstrated that IL-2 is an important signal for the development, function and homeostasis of natural T_regs_ *in-vivo* [22]–[29]. Tang et al.[30] reported that in the NOD mice (which spontaneously develops diabetes), disease progression was associated with a loss of T_reg_: CD4^+^T_eff_ balance in the islets with concomitant reduction of *CD25* and *BCL2* expression on intra-islet T_reg_ cells. Addition of IL-2 promoted T_reg_ cell survival and protected NOD mice from diabetes. Further, in the NOD mouse, Yamanouchi et al. have shown that IL-2 gene variation impairs T_reg_ function and influences diabetes susceptibility. They have also shown that the amount of IL-2 produced by autoreactive CD8+ T cells in response to antigen seems to control the size of the lymph node T_reg_ pool [31]. Based on this collective evidence of the role of IL-2 in T_reg_ function, we hypothesized that reduced IL-2 could be partially responsible for the increased apoptosis and reduced function of T_regs_ from human T1D subjects. Further evidence in support of this hypothesis is presented in the following sections.

### Increased apoptosis in T_regs_ from T1D subjects is reproducible in healthy T_regs_, under conditions of IL-2 deprivation

**Figure 2:**
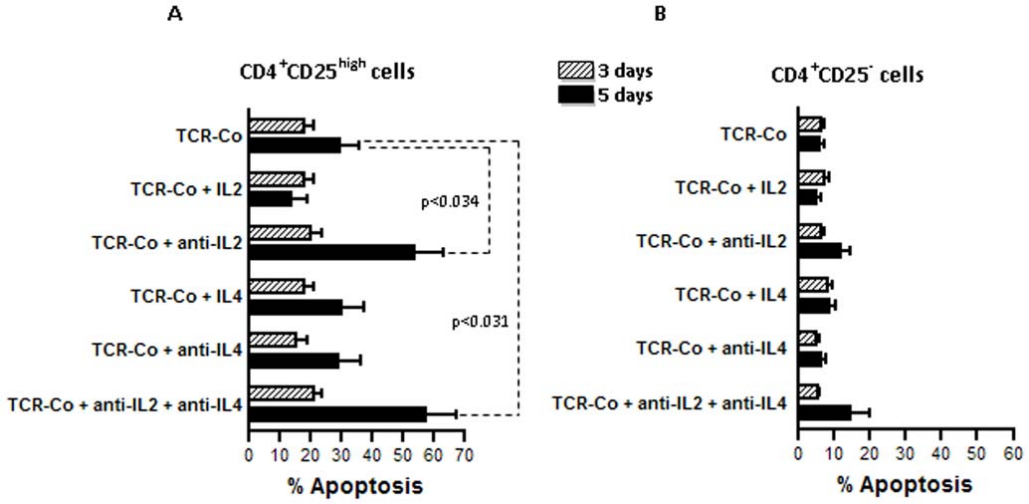
Under conditions of IL-2 and IL-4 withdrawal, apoptosis in healthy T_regs_ mimics the increased apoptosis in T_regs_ from T1D subjects. Apoptosis was measured in (A) healthy T_regs_ and (B) CD4^+^CD25^−^ effector T-cells at 3 days and 5 days, in response to IL-2 and IL-4 deprivation as described in the methods. Overall, in response to IL-2- and IL-4- deprivation, T_regs_ show more apoptosis compared to effector T-cells. Further, within T_regs_, withdrawal of IL-2 leads to a strong apoptosis response, compared to withdrawal of IL-4. Also, this difference in apoptosis was more prominent after 5 days of IL-2 withdrawal. The bars depict the mean±SEM of six independent observations.

First, we tested if increased apoptosis could be reproducible in healthy T_regs_, under conditions of IL-2 and IL-4 deprivation. It can be argued that it is more useful to establish the link between cytokine deprivation and apoptosis directly in T_regs_ from T1D subjects. However, functionally deficient T_regs_ from T1D subjects are likely to undergo irreversible apoptosis/necrosis in response to IL-2 deprivation, making this sort of analysis uninformative. Apoptosis was measured in T_regs_ and in CD25^−^ T_eff_ cells from control subjects, at 3 days and 5 days after IL-2, IL-4 and IL-2+IL-4 withdrawal. Results show that there was increased apoptosis in T_regs_, but not in CD25^−^ T_effs_ in response to IL-2 and IL-4 deprivation (Figure 2). Further, within T_regs_, withdrawal of IL-2 leads to a strong apoptosis response, compared to withdrawal of IL-4. Also, at 5 days of co-culture, the effect of IL-2-deprivation on apoptosis was more pronounced. Remarkably, this pattern of elevated apoptosis in healthy T_regs_ under conditions of IL-2 deprivation replicated that observed in T_regs_ from T1D subjects when compared to control subjects.

### Gene expression signatures in T_regs_ from T1D subjects reveal modulation of several apoptosis genes, possibly due to cytokine deprivation

We then proceeded to profile gene expression changes that could discriminate functionally deficient and apoptosis-sensitive T_regs_ from their healthy counterparts. Out of 16 T1D and 25 control subjects assayed for T_reg_ apoptosis and suppression, 12 T1D and 15 control subjects yielded sufficient RNA for expression profiling. T_regs_ were freshly isolated from PBMC and amplified RNA was hybridized to the microarray as described in the methods. Bayesian hierarchical analysis identified 1205 differentially regulated genes (|z-score|>20) across the two phenotypic groups. Of these, 947 genes were upregulated in T1D subjects and 248 genes were downregulated in T1D subjects, compared to control subjects. T_regs_ used in this expression study were not subjected to any stimulation, as these cells are known to be susceptible to activation-induced cell death, whereby any resulting differences in apoptosis-related gene expression could be nullified [32], [33]. Hence the relatively modest magnitude of expression changes (±2.5-fold or less) was expected in this dataset. It is now known that for minimal changes in gene expression, instead of a gene-by-gene approach [34], detecting predefined co-regulated gene sets for association with disease phenotypes has more power of discovering biological themes within functionally related sets of genes. We used EASE, a pathway enrichment tool, to find the biological pathways perturbed in each phenotype [35]. Within EASE, three functional categorization systems, viz. Organismal role, GO Molecular function and Interpro were probed individually for pathway terms associated with each of the two phenotypes. To rank pathway terms by over-representation, we used the significance cutoffs on the EASE score (see the methods section for details on the EASE score). The 5 most significantly enriched pathway terms and associated EASE scores under each of the three categorization systems are shown in Table 1. For the gene list downregulated in T1D subjects, there was a near-significant enrichment (EASE score<0.06) of terms related to *cell migration/motility*, *cytokine binding, MHC class II receptor activity, chemokine receptor activity*, *chemokine receptor & MHC Class II, alpha chain*.

**Table 1:** Pathway enrichment analysis using EASE.

	EASE score	Gene Category	EASE score
**Organismal role**		**Organismal role**	
General cellular role	3.73E-05 *	Anti-pathogen response	2.92E-02
Angiogenesis	8.77E-02	Cell migration/motility	5.83E-02
Cell death/Apoptosis	1.18E-01	Pathogenic Invasion	6.68E-02
Anti-pathogen response	1.40E-01	Neuronal development	3.01E-01
Control of Cell Proliferation	5.47E-01	Other development	6.35E-01
**GO Molecular Function**		**GO Molecular Function**	
Nucleic acid binding	9.92E-08 *	Cytokine binding	9.00E-05
Hydrogen ion transporter activity	1.16E-07 *	MHC class II receptor activity	1.28E-04
Monovalent inorganic cation transporter activity	3.70E-07 *	Transmembrane receptor activity	1.34E-04
RNA binding	2.13E-06 *	C-C chemokine binding	2.46E-04
Cation transporter activity	3.02E-05 *	C-C chemokine receptor activity	2.46E-04
**Interpro**		**Interpro**	
Helicase C-terminal domain	1.08E-02 *	Chemokine receptor	5.93E-03
DEAD/DEAH box helicase	1.08E-02 *	Immunoglobulin C-type	2.94E-02
ATP-dependent helicase, DEAD-box	1.80E-02 *	MHC Class II, alpha chain	3.21E-02
Proline rich extensin	3.52E-02 *	Ras GTPase-activating proteins	7.33E-02
Spectrin repeat	6.44E-02	Immunoglobulin and MHC domain	1.12E-01

Left panel): Top 5 terms amongst the 948 genes upregulated in T1D (FDR<5%), in each of the three functional classification systems. (Right panel): Top 5 terms amongst the 257 genes downregulated in T1D (FDR<5%), in each of the different functional classification systems. Terms marked with an asterisk (*) are statistically significant at EASE score <0.05

**Figure 3:**
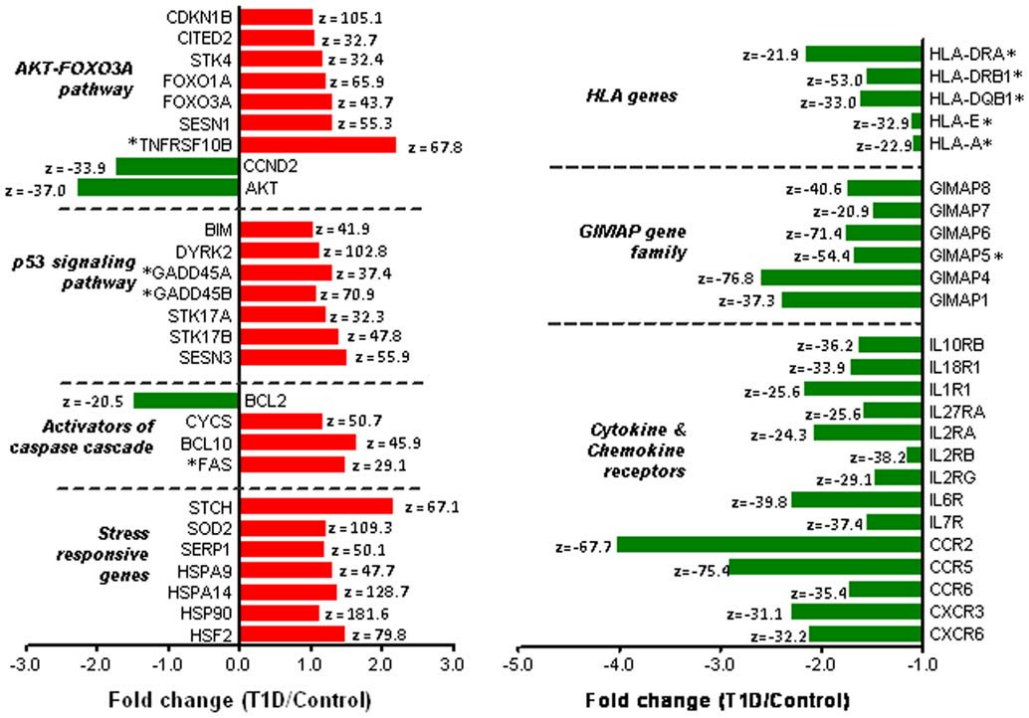
Expression trends of differentially regulated apoptosis genes on the array. This panel of genes shows the identity, functional grouping and the fold change of genes differentially regulated across T1D and control subjects (|z-score|>20). The red bars represent genes that are upregulated in T1D subjects compared to controls and the green bars represent genes that are downregulated in T1D subjects compared to controls. The fold changes are as reported by BGX analysis. The gene symbols with an asterisk (*) represent genes whose expression was confirmed on the microarray as well as by RT-PCR.

Next, we extracted the identities and annotations of the genes that featured in the enrichment results, as well as other differentially expressed genes that are members of these functional clusters. These differentially regulated genes are shown in Figure 3, along-with their functional grouping and associated fold changes. Our primary observation was that several apoptosis-related genes were differentially regulated in T_regs_ from T1D subjects, compared to control subjects (all significant at |z-score|>20). *BCL2*, an anti-apoptotic gene which represses the caspase cascade and is known to make CD4^+^ T cells sensitive to apoptosis under conditions of serum starvation [36], was downregulated in T1D subjects. Cytochrome c (CYCS), whose release from the mitochondria is normally blocked by *BCL2*, as well as *FAS* and *BCL10*- two major caspase activators, were also upregulated in T_regs_ from T1D subjects. Further, genes related to stress response and known to be triggered by lack of survival factors [37], were upregulated in T_regs_ from T1D subjects. The transcription factor *FOXO3A* as well as some its key pro-apoptotic transcriptional targets (*GADD45A, GADD45B, SESN1, CDKN1B, CITED2* and *TNFRSF10B*) were upregulated in T_regs_ from T1D subjects. Pro-apoptotic genes in the *p53* signaling pathway (*STK17A, STK17B, GADD45A, GADD45B, SESN3, DYRK2* and *BIM*) were upregulated in T_regs_ from T1D subjects. Finally, several members of the GIMAP gene family (implicated with T1D in the rat [38]–[40]), HLA genes as well as cytokine/chemokine receptors were downregulated in T_regs_ from T1D subjects. In summary, the downregulation of AKT1 and the concomitant upregulation of FOXO3A/p53 targets in T_regs_ from T1D subjects mirrored a cytokine deprivation mediated stress response that could lead to AKT pathway mediated cell cycle arrest.

**Figure 4:**
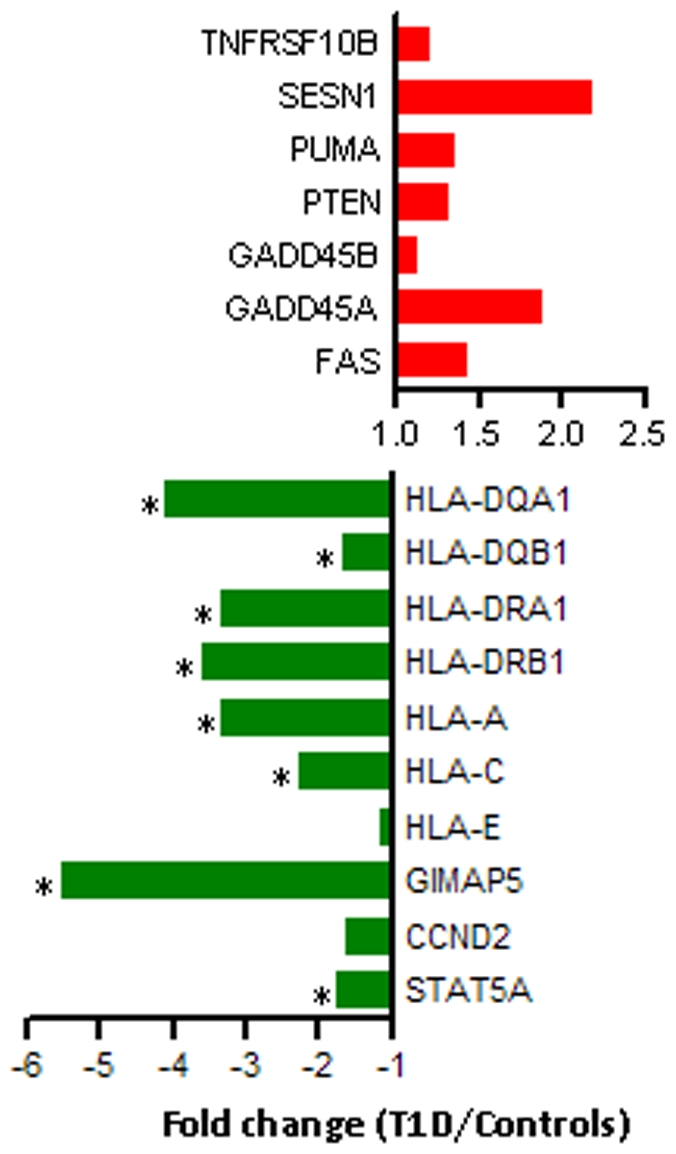
Confirmation of array results by RT-PCR. Expression results for several genes in the AKT pathway and HLA genes were confirmed by RT-PCR. For genes in the AKT pathway, the expression value is a ratio of the average expression across T1D subjects (n = 5) to the average expression across control subjects (n = 5). For HLA Class II genes, the expression value is a ratio of the average expression across T1D subjects (n = 6) to the average across controls subjects (n = 8). For HLA Class I genes, the expression value is a ratio of the average across T1D subjects (n = 10) to the average across control subjects (n = 10). Genes for which the expression ratios were statistically significant (Mann-Whitney p≤0.05) are marked with an asterisk (*). Subjects are not the same as those used for expression profiling.

For confirmation of array results by quantitative RT-PCR, we restricted the selection of apoptosis-related and *HLA* Class I/Class II genes to only those that were reported as differentially expressed using both SAM (FDR<10%) and BGX (|z-score|>20) analyses. RT-PCR results (Figure 4) confirm the upregulation of *PTEN*, *FAS*, as well as several pro-apoptotic transcriptional targets of *FOXO3A* in T_regs_ from T1D subjects (*GADD45A, GADD45B, SESN1, PUMA* and *TNFRSF10B*). Downregulation of *CCND2*, a negative target of *FOXO3A*, was also confirmed. Trends in the expression of HLA Class I (*HLA-A, HLA-C, HLA-E*) and HLA Class II genes (*HLA-DQA1, HLA-DQB1, HLA-DRB1, HLA-DRA1*) were similar to those observed on the array. In agreement with the array results, *GIMAP5* was also found to be downregulated by RT-PCR. Thus, RT-PCR results confirm several array results, and establish the expression changes in the *AKT* pathway that make T_regs_ from T1D subjects sensitive to apoptosis.

**Figure 5:**
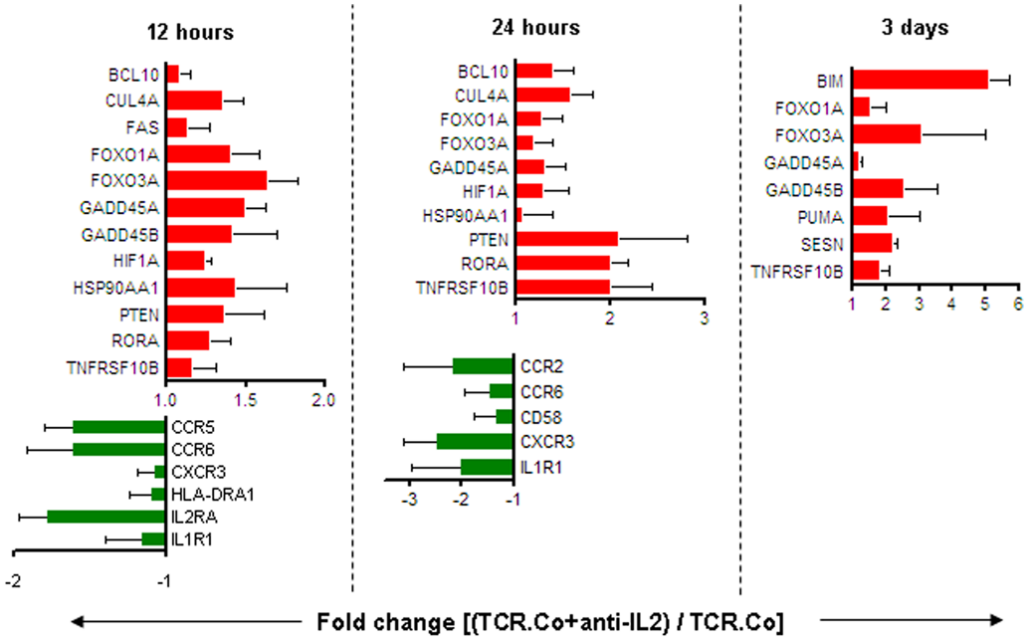
Under condition of IL-2 withdrawal, expression changes of key apoptosis and cytokine/chemokine genes in healthy T_regs_ are similar to those observed in T_regs_ from T1D subjects. Expression changes in 32 selected genes were captured in healthy T_regs_ treated with aCD3+aCD28 with or without anti-IL-2, for indicated time points. The figure shows a subset of apoptosis and cytokine/chemokine receptor genes, whose expression trends in IL-2-deprived healthy T_regs_ were similar to those in T_regs_ from T1D subjects. Red bars indicate genes upregulated on IL-2 deprivation and green bars indicate genes downregulated on IL-2 deprivation in healthy T_regs_. The bars depict the mean±SEM of three independent observations.

For reproducing expression signatures seen in T_regs_ from T1D subjects, we selected 32 key genes from the various functional clusters discussed so far (15 apoptosis-related genes, 11 cytokine & chemokine receptors and 6 GIMAP genes), and checked their expression trends in healthy T_regs_, under conditions of IL-2 deprivation. As array results suggested early gene expression changes leading to apoptosis, we checked RNA expression at 12 hours, 24 hours and 3 days after IL-2 withdrawal. Results show that expression trends in TCR-Co (αCD3+αCD28) stimulated healthy T_regs_ under IL-2 withdrawal were in agreement with expression trends in T_regs_ from T1D subjects, for 19 of the 32 assayed genes (Figure 5). Expression trends in key apoptosis genes along the AKT/p53 signaling axis (upregulation of transcriptional targets of *FOXO3A*: *TNFRSF10B, GADD45A, GADD45B* and *FAS*) and cytokine/chemokine receptors (downregulation of *IL1R1, CCR2, CCR6, CD58* and *CXCR3*) mimic those observed in T_regs_ from T1D subjects, at one or more time-points. Further, in the gene-expression results, important cytokine-responsive lymphocyte activation markers: interleukin-2 receptor alpha (*IL2RA*), chemokine (C-C motif) receptor 5 (*CCR5*) and human leukocyte antigen, class II, DR alpha 1 (*HLA-DRA1*) were downregulated in T_regs_ from T1D subjects. It has been documented that these three activation-linked genes are positively regulated by IL-2 in various T-cell subsets [41], [42]. In line with our hypothesis, withdrawal of IL-2 resulted in reduced expression of these cytokine-responsive genes.

**Figure 6:**
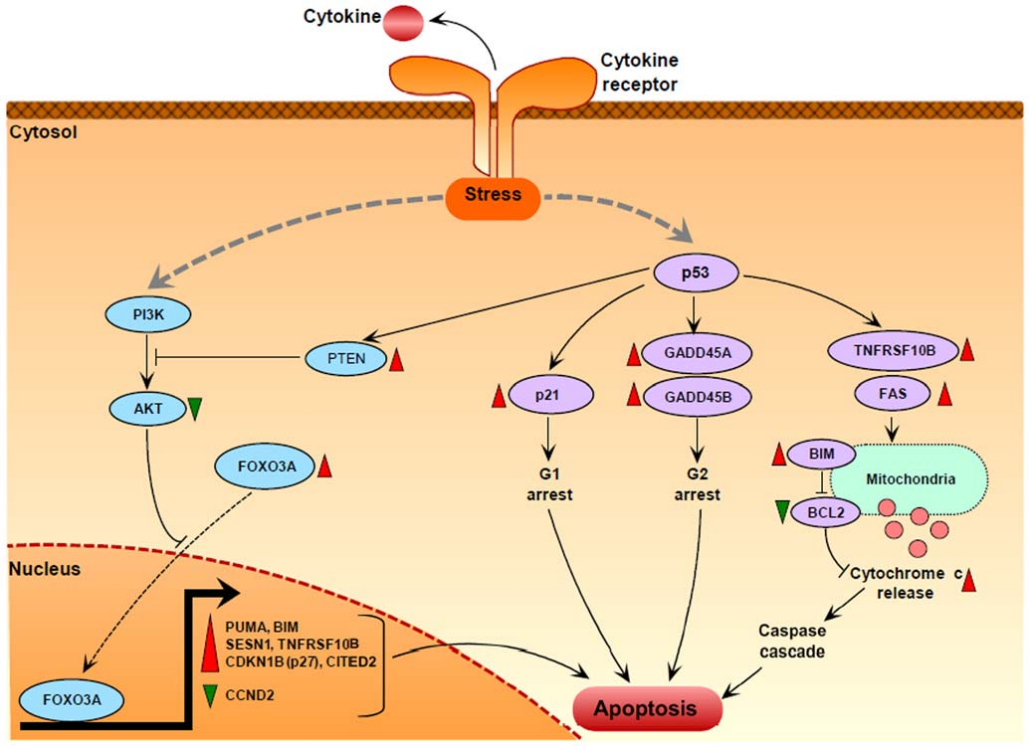
Putative cytokine deprivation mediated apoptosis signaling pathways in T_regs_ from T1D subjects. In the absence of cytokines or growth factors, the stress-induced PI3K-AKT pathway and the p53 pathway are activated. Downregulation of PI3K and AKT through inhibition by PTEN leads to translocation of dephosphorylated FOXO3A into the nucleus. Nuclear FOXO3A activates several pro-apoptotic genes (PUMA, BIM, SESN1, TNFRSF10B, CDKN1B, CITED2) and represses CCND2 (a cell cycle gene) by direct transcriptional control. Modulation of these FOXO3A targets then leads to cell cycle arrest and apoptosis. On the other hand, activation of p53 further represses AKT via induction of PTEN, and also leads to a cascade of expression changes in several apoptosis genes. Activation of p21 and the two GADD45 genes leads to cell cycle arrest at the G1 and G2 phases respectively. Ligation of TRAIL with TNFRSF10B (TRAILR) and activation of FAS on the surface of T_regs_ triggers the extrinsic apoptosis pathway, leading to the release of cytochrome c from the mitochondria and activation of the caspase cascade. This complex signaling involving cytokine-deprivation mediated activation of both the intrinsic pathway as well as the extrinsic pathway leads to increased T_reg_ apoptosis in T1D subjects. Genes with red triangles were upregulated, while genes with green triangles were downregulated in T_regs_ from T1D subjects (|z-score|>20 on the array). This pathway diagram is constructed using known gene-gene interactions from the literature, for those genes that were discussed in this study either on the array, RT-PCR or under conditions of IL-2-deprivation.

In summary, expression trends in healthy T_regs_ under cytokine deprivation were identical to those observed in T_regs_ from T1D subjects. These lines of evidence support the hypothesis that the expression signature in T_regs_ from T1D subjects may be partially induced by a cytokine deficient milieu. A pathway diagram in Figure 6 captures the multiple signaling events that could trigger apoptosis in T_regs_ from T1D subjects, under conditions of cytokine deprivation.

## Discussion

IL-2, a T-cell growth factor, plays an important role in multiple aspects of T-cell biology. Apart from the effect of IL-2 on T_reg_ activation, there is strong evidence supporting a role for IL-2 in the development and/or function of T_regs_ [25], [26], [43]. In close proximity to T_regs_ *in vivo*, are effector T-cells which control T_reg_ development and function by secreting several cytokines, including IL-2. There is evidence for an imbalance in cytokine secretion from effector cells in T1D [44]. In the murine TS1αβ cell line, following IL-2-deprivation, there was decreased proliferation and increased apoptosis over time [45]. Some studies have reported decreased IL-2 production in recent-onset T1D patients and low IL-2 production persisting for years after the onset of disease [46], [47]. T_reg_ stimulation occurs *in vivo* by cytokines secreted from various effector cell types, but the CD4+ effector T cells which are the major producers of IL-2, play a primary role in shaping the T_reg_ maturation and response. We also compared the production of cytokines in both the CD4^+^CD25^−^ and the CD4^+^CD25^low^ effector T-cell subsets from T1D and control subjects. As shown in Figure S3, for both these effector T-cell subsets, after 12 hours of αCD3 and αCD28 stimulation, production of some important cytokines (IL-2, IL-4, IL-5, IL-10, IFNγ and TNFα) was reduced in T1D subjects compared to control subjects, with the maximum difference in levels of IL-2 secretion (p<0.05 for IL-2, CD4^+^CD25^−^). Further, although differences in the production of other cytokines across the two groups were not statistically significant, the trends were similar for each of the measured cytokines. These results collectively suggest that increased apoptosis, decreased T_reg_ function and concomitant gene expression changes could be linked to deficient IL-2 secretion from effector T-cells.

In line with this hypothesis, we observed elevated T_reg_ apoptosis and reduced T_reg_ suppressive function at the onset of disease. Considering that T_regs_ are selective targets for destruction, expression profiling within T_regs_ provided us with a model for understanding the cause (T_reg_ environment) by studying the effect (T_reg_ expression changes). Our data supports the notion that in patients with T1D, the lack of suppression of autoreactive T-cells in the periphery is a result of increased apoptosis in T_regs_, possibly precipitated by deprivation of growth signals such as IL-2. Increased expression of various apoptosis genes, and the downregulation of pro-survival *GIMAP* genes indicate a complex interplay amongst these pathways leading to apoptosis. Further, co-ordinate repression of cytokine receptors and HLA genes points to a possible proliferation defect in T_regs_ from T1D subjects.

The *FOXO* subfamily of forkhead transcription factors plays an evolutionarily conserved role in cellular adaptation to stress stimuli and also regulates survival in response to cytokine deprivation, DNA damage and oxidative stress. Amongst several reports documenting apoptosis of lymphoid cells under conditions of cytokine deprivation [48], [49], there is increasing evidence for the involvement of the PI3K/AKT and the closely intertwined p53 signaling pathways [50]–[53]. Further reports have shown that under conditions of cytokine deprivation mediated cellular stress, AKT-mediated activation of FOXO3A and subsequent transcriptional regulation by FOXO3A leads to cell cycle arrest and apoptosis [54], [55]. In T_regs_ from T1D subjects, we observe *AKT* to be downregulated, while *PTEN* and *FOXO3A* are upregulated, suggesting that in the absence of activated *AKT, FOXO3A* phosphorylation may be inhibited, which could lead to its activation and translocation to the nucleus. Transcriptionally active *FOXO3A* induces several pro-apoptotic genes such as *PUMA, BIM, GADD45A, GADD45B, SESN1, TNFRSF10B, CITED2* and *CDKN1B*, all of which were upregulated in T_regs_ from T1D subjects (significant either on the array at |z-score|>20 or on RT-PCR at p<0.05) as well as show similar trends in healthy T_regs_ under conditions of IL-2 withdrawal. Our data suggests that in T_regs_ from T1D subjects, both the AKT and p53 pathways exert pro-apoptotic function through transcriptional regulation of multiple downstream targets that render the cells sensitive to apoptosis.

Few studies have reported on the expression of HLA molecules in T-cells of patients with T1D. HLA class I expression has been reported to be decreased [56] or normal [57], [58], whereas HLA class II expression on the surface of PBMC of T1D patients has been reported to be normal [56]. Reduced expression of HLA Class I as well as Class II molecules in freshly isolated T_regs_ from T1D subjects could lead to defects in HLA-TCR interactions between T_regs_ and various other T-cell subsets in the periphery. Recently, it has been demonstrated that the direct *ex vivo* expression of HLA-DR in the context of CD4^+^CD25^high^ T cells identifies a mature, functionally distinct regulatory T cell population involved in contact-dependent in vitro suppression[59]. Our observation of reduced HLA Class II in T_regs_ from T1D subjects and the associated loss of suppressive capacity is in line with this report. We are currently following up this relationship across HLA expression, T_reg_ function and genotype as a separate study.

Several genes in the *GIMAP* family of novel GTPases (*GIMAP4, GIMAP5, GIMAP1, GIMAP7* and *GIMAP8*) are also downregulated in T_regs_ from T1D subjects. Recent reports suggest crucial roles of *GIMAP* family proteins in regulating T-cell development, selection and homeostasis [60], [61], and GIMAP4/5 have been shown to interact with *BCL2* family members, thereby regulating T-cell survival [39]. GIMAP5 gene mutations have been shown to contribute to T1D in the rat model for diabetes [62], [63]. As the downregulation of GIMAP genes in T_regs_ from T1D subjects could not be reproduced in healthy T_regs_ under IL-2 withdrawal, the IL-2 pathway does not seem to control expression of GIMAP genes.

It is interesting to observe that some of the differentially expressed genes in this study have been discussed in other T_reg_ gene expression studies, in both the physiological and disease contexts. Amongst a subset of direct FOXP3 targets that exhibited consistent transcriptional behavior in hybridomas and in *ex vivo* T-cells [64], we found several negative targets of FOXP3 (*EVI2B, GADD45B, PTPN22, TGIF, MYC, PHF6, POU2AF1 and GPR171*) to be upregulated in T_regs_ from T1D subjects while positive FOXP3 targets (*IL2RA, CD2 and CPEB2*) were downregulated in T_regs_ from T1D subjects. In summary, our results provide evidence that dysregulation in T_reg_ specific as well as FOXP3 associated genes could lead to functionally compromised T_regs_ in T1D subjects.

Our study design has some limitations. First, Our T_reg_ isolation strategy may not be the most optimal way to isolate pure T_regs_. However, isolation of bonafide regulatory T-cells remains difficult because the availability of specific marker molecules is still limited. Apart from CD25, additional surface molecules have been proposed as useful markers to distinguish regulatory from effector T-cells, such as CTLA4, TNFRSF18 (GITR), CD62L and NRP1. However, many of these molecules are also expressed by naïve CD4^+^CD25^−^ T-cells upon activation, thereby hampering discrimination between regulatory and conventionally activated CD4^+^ T-cells. Here we adopted a very conservative T_reg_ isolation strategy, selecting only the top 1% CD25 expressing cells as T_regs_. The additional confirmation that most of these cells were CD127-ve ensures that our cell population is highly enriched for T_regs_ (Figure S4-A). Second, subjects in both groups belonged to different age groups. However, we have earlier shown that the apoptosis of T_regs_ is not related to age effects [65]. Third, although it would be more useful to confirm the results on apoptosis and cytokine receptor genes directly on T_regs_ from T1D subjects, we had to use healthy T_reg_ blood-donor subjects for lack of adequate numbers of fresh T_regs_ from children with T1D. Further, subjecting T_regs_ from T1D subjects to IL-2 deprivation would have not have much use, as they are already in an environment of cytokine deprivation. Nevertheless, the expression of pro-apoptotic genes in T_regs_ from healthy subjects under conditions of IL-2 deprivation mimicked the behavior of the same genes in T_regs_ from T1D subjects, which gives indirect yet convincing evidence in support of our hypothesis. Lastly, with this study design, it is not possible to determine precisely the extent to which the expression profile associated with the T1D phenotype could be mediated by possible secondary effects of insulin or hyperglycemia. However, we have previously shown that long-standing T1D subjects as well as longstanding T2D subjects who are also on insulin do not show the same T_reg_ defects as in the at-risk or recent-onset T1D subjects [10]. Hence, it is unlikely that there is any significant effect of exogenous insulin or hyperglycemia on T_reg_ apoptosis.

To conclude, this report establishes the expression signature within T_regs_ in the periphery, during the period right after the clinical onset of T1D. T_regs_ in T1D subjects express several pro-apoptotic genes, specifically along the AKT- and p53- signaling axis, which may be partially a result of cytokine deprivation. This identification of a molecular signature of T_regs_ during the recent-onset T1D period is an important step in understanding T_reg_ action and could serve as an important diagnostic for T_regs_ that have lost the potential to suppress autoreactive effector cells. Understanding the mechanism by which cytokine deprivation in T1D induces expression of apoptotic genes will reveal novel points along the death-promoting cascade, which can be targeted for therapeutic interventions.

## Materials and Methods

### Ethics Statement

The research protocol was approved by the Institutional Review Board (IRB) of the Children's Hospital of Wisconsin IRB no. 01–15 and participants and/or their parents (guardians) provided written informed consent.

### Human subjects and clinical measurements

Recent-onset T1D subjects (after stabilization on exogenous insulin but within 12 months of diagnosis; n = 15) were recruited through the diabetes program at Children's Hospital of Wisconsin. Diabetes was defined according to World Health Organization criteria and included blood glucose levels of >200 mg/dl with symptoms confirmed by a physician [66]. Healthy control (n = 17) subjects were recruited by posting flyers in Children's Hospital of Wisconsin and the Medical College of Wisconsin. The control criteria comprised fasting blood glucose of <100 mg/dl, no familial history of any autoimmune disorder, and negativity for islet autoantibodies at the 99th percentile. All study subjects were free of known infection at the time of sample collection. At the time of each visit, the following clinical measurements were taken: HbA1c, glucose level, height, weight and BMI (subject characteristics are shown in Table 2). The presence of autoantibodies was measured from peripheral blood.

**Table 2:** Subject demographics and clinical measurements.

	Recent-onset T1D	Healthy Controls
Number of subjects	12	15
Gender	4M/8F	5M/7F
Age at visit (years)	13±5	35±14
Height (cm)	153±19	169±9
Weight (kg)	47±18	67±14
BMI	19±3	23±3
Glucose (mg/dl)	203±79	87±12
Duration of T1D (months) (range: 0.48–10.56)	4.4±3.3	NA
HbA1c (%)	7±0.8	NA
Insulin dose (ave.units/kg/day) (range: 0.05–0.75)	0.46±0.27	NA
NA = Not Applicable		

### CD4^+^CD25^high^ T-cell isolation

As this study assessed whether alterations in the function of regulatory T-cells could be involved in the pathogenesis of T1D, it was crucial to identify highly pure and homogeneous regulatory T-cells. It is now known that while the entire population of CD4^+^CD25^+^ T-cells, expressing both low and high CD25 exhibits regulatory function in the mouse, only the CD4^+^CD25^high^ population exhibits a similarly strong regulatory function in humans [67]–[69]. Using the same FACS isolation protocol described earlier [10], we collected the top 1% of CD4^+^CD25^high^ T-cells as T_regs_ for this study. CD4^+^CD25^low^ and CD4^+^CD25^−^ effector T cells were also isolated using the same protocol. This additional stringency of collecting just the top 1% of CD4^+^CD25^high^ cells as T_regs_ ensured removal of most of the activated CD25^low^ T-cells. We found that most of the T_regs_ we isolate are CD127-ve [70], [71], while most of the CD4^+^CD25^−^ cells are CD127^+^, as an added confirmation of the purity of the isolated T_regs_. See Supporting Fig. S5-A for a representative plot of this standardized sorting procedure. Further, as shown in Figure S4-B, intracellular staining for FOXP3 positivity also showed that T_regs_ isolated using this protocol had a significantly higher percentage of FOXP3^+^ cells (61.88±2.68) compared to CD4+CD25̅ cells (4.32±0.80) (Mann-Whitney p<0.0001). Isolated T_reg_ cells were anergic and showed good suppressive capacity *in vitro* (data not shown). Thus, our isolation protocol generated T_regs_ that maintained high level of sustained FOXP3 expression associated with phenotypic and functional stability.

### RNA extraction and hybridization

Total RNA was extracted from CD4^+^CD25^high^ T-cells using TRIzol Reagent (Invitrogen Life Technologies) according to the manufacturer's protocol. The GeneChip human genome U133 plus 2.0 array (Affymetrix) was selected for this study which interrogates >47,000 probe sets, representing roughly 39,000 unique genes. Purified total RNA (∼50 ng) was amplified using an Affymetrix two-cycle cDNA synthesis kit (catalog no. 900432) and cRNA was synthesized, labeled, fragmented and hybridized to the arrays in accordance to standard Affymetrix protocols. After hybridization arrays were washed, stained with PE-conjugated streptavidin (Molecular Probes) and scanned on a GeneChip Scanner 3000 (Affymetrix). Image data were analyzed with Affymetrix GeneChip operating software (GCOS).

### Microarray quality control

Each microarray scan was visually inspected for irregularities, and the quality of the entire microarray set was assessed using the *affyQCreport* package from the Bioconductor project [72]. Information from the various QC plots (Figure S5) confirmed good data quality across arrays.

### Bayesian hierarchical analysis

Most existing methods for studying differential expression adopt a stepwise procedure for obtaining a point estimate of expression for each gene on each array. Having obtained point estimates for all the genes, studies of differential expression between pairs of phenotypes are carried out by comparing the collections of point estimates under the phenotypes, using a t-type statistic such as in SAM [73], Cyber-T [74] or Limma [75], all of which rely on the availability of replicate subjects in each phenotypic condition, for estimation of variances. These approaches are not feasible when the number of subjects is low. As limitations on the amount of available RNA restricted our analysis study to a limited number of subjects in each phenotype, we used Bayesian Gene eXpression, BGX [76]-[78], which implements a statistical procedure that works with minimal or no replicates in each phenotypic group. The BGX method differs from these stepwise point estimate approaches in that (1) uncertainties associated with each of the steps are taken into account at all levels of analysis, (2) gene- and phenotype- specific expression levels are estimated from a joint consideration of the available probe set intensities and (3) the outcomes are posterior distributions of expression rather than point estimates. A flowchart for all steps of this analysis is shown in Figure S6. The program was executed with the following parameter values: number of probes: 604258; number of genes: 54675; number of categories: 1, number of probes with no sequence information: 0; number of genes to monitor fully: 0; sub-sampling interval: 16; number of burn-in sweeps: 8192; number of sampling sweeps: 16384; spread of jumps in S: 30; spread of jumps in H: 350; spread of jumps in mu: 1.1; spread of jumps in sigma: 1.5; spread of jumps in lambda: 0.04; spread of jumps in eta: 0.1; batch size: 50 and optimal acceptance ratio: 0.44. Briefly, in the first step, a Bayesian hierarchical model is formulated for estimating expression levels from probe level expression data from all the arrays. For each gene in each phenotype (phenotype 1: T1D and phenotype 2: Control), the gene expression index *μ*_g1_ (level of expression of gene _g_ in T1D) and *μ*_g2_ (level of expression of gene _g_ in Controls) is calculated. Within this framework, the samples from the posterior distributions of the differences (*d_g_* = *μ_g,1_ − μ_g,2_*, *g = 1,…54675*) represent a natural base for inference on differential expression. For each gene, a kernel density plot for the expression index *μ_gp_* and the difference *d_g_* was constructed. From the cumulative information of expression differences (*d_g_*, g = 1 to 54675), a histogram of the posterior probabilities of expression differences being less than zero [*P(d_g_<0)*,g = 1, …54675] is determined (Figure S7). The null distribution is estimated by spline fitting [79] on the histogram, and the difference between the histogram fit (grey curve) and the null distribution (black curve) is used to give an estimate of the number of differentially expressed genes. An excess of *P*(*d_g_*<0) values in the left as well as right tails indicates the presence of both over- and under-expressed genes respectively. In the final step, genes are ranked using the standardized BGX difference (z-score) between the two conditions, which takes into account the estimated difference in expression level as well as the associated uncertainty. The z-score also takes into account the autocorrelation structure of the sequence of values generated by the algorithm, whereby genes with highly autocorrelated values (high variability) are automatically given lower ranks, thereby increasing the power to detect differential expression. The identities of the differentially expressed genes are found by selecting from each end of the ranked list, the number of differentially expressed genes predicted to be in the tails of the histogram of posterior probabilities. The analysis predicted a total of 1205 differentially expressed genes, of which 948 genes were upregulated in T1D (left tail of the histogram) and 257 genes were downregulated in T1D (right tail of the histogram). Although rank thresholds from the histogram were used instead of an arbitrary z-score threshold for picking differentially expressed genes, all genes within the list of 1205 differentially satisfied the |z-score|>20 threshold, which has been shown to yield a low <5% FDR on the Golden spike control dataset [76]. There was a 78% overlap among differentially expressed genes predicted through BGX (FDR<5%) and SAM (t-test, FDR<9.5%), which verifies the predictive accuracy of this new Bayesian technique, when compared to a frequentist approach. These data have been deposited in NCBI's Gene Expression Omnibus (GEO, http://www.ncbi.nlm.nih.gov/geo/) and are accessible through GEO Series accession number GSE10586.

### Gene annotation and pathway enrichment analysis

Gene annotation was carried out using controlled vocabularies from the Gene Ontology database [80]. Information from gene-gene interactions on multiple KEGG pathways (http://www.genome.jp/kegg) was assimilated for constructing the putative signaling diagram shown in Fig. 6. Pathway enrichment analysis was carried out using EASE [35]. Briefly, in EASE, the following terminology is used for pathway enrichment analysis: List Hits = number of genes in the gene list that belong to the Gene Category; List Total = number of genes in the gene list; Population Hits = number of genes in the total group of genes assayed that belong to the specific Gene Category; Population Total = number of genes in the total group of genes assayed that belong to any Gene Category within the System. The first step is the mapping of primary gene identifiers to gene categories within various categorical systems. Then, the “Population Total” is determined for each system of gene categorization, and the “Population Hits” is determined for every category within those systems. Now given a gene list that represents some sub-set of the population genes, the “List total” and “List Hits” counts can be determined. The probability of seeing the number of “List Hits” in the “List Total” given the frequency of “Population Hits” in the “Population Total” is calculated as the EASE score. The EASE score is essentially a sliding-scale, conservative adjustment of the Jackknife Fisher exact probabilities that strongly penalizes the significance of categories supported by few genes and negligibly penalizes categories supported by many genes, therefore yielding more robust results.

### Real-Time PCR analysis

Total RNA (25 to 50 ng) was converted to cDNA using the QuantiTect® reverse transcription kit (Qiagen). Real-time PCR was then performed using the QuantiTect® SYBR Green PCR Kit (Qiagen) on an ABI Prism 7900HT sequence detection system machine with SDS software (Applied Biosystems). Manufacturer protocols were followed for all procedures. mRNA gene-expression was quantified relative to either GAPDH or 18 s rRNA housekeeping genes. Using the Oligo 6 software (Molecular Biology Insights), primer sets for each gene were designed towards the same region of cDNA that was represented by the probe sets on the Affymetrix GeneChip arrays.

### Apoptosis assay

Apoptosis was measured using a dual-staining method that our group has reported to be very sensitive for apoptosis detection [81]. Briefly, 15,000–25,000 CD4^+^CD25^high^ T-cells were stained with YOPRO1 (250 nM) for 20 minutes in the dark and 7AAD (250 ng) was added 10 minutes before acquiring events on a FACS LSRII (BD Biosciences). The data was then analyzed using the FACSDiva Software (BD Biosciences). Apoptosis was measured as the percentage of apoptotic cells YOPRO1+ve/7AAD-ve amongst live cells (total 7AAD-ve cells comprising both YOPRO1+ve and YOPRO1-ve cells).

### Suppression assay

CD4^+^CD25^−^ T-cells (2.5×10^4^ cells/well) were cultured in RPMI 1640 media supplemented with 2 mM L-glutamine, 5 mM HEPES, 100 U/µg/ml penicillin/streptomycin, 0.5 mM sodium pyruvate and 10% human AB serum. Cells were stimulated with αCD3 coated beads (1 µg/ml, 3 beads/cell) in U-bottom 96 well plates (Costar) in the presence of the same number of irradiated autologous PBMC for 5 days. For the suppression assays CD4^+^CD25^high^ T-cells were co-cultured with CD4^+^CD25^−^ at a 1∶10 ratio (T_reg_:CD25^−^ T_eff_) using the same stimuli. Cells were pulsed with 1 µCi of [^3^H] thymidine (Amersham Pharmacia Biotech) and harvested after 16 hours. The cpm per well was determined with a scintillation counter (Top Count NXT, Packard). The percentage of suppression was calculated as {(s−c)/s}×100%, where s = cpm in single culture and c = cpm in co-culture.

### Intracellular Staining for FOXP3

Intracellular staining for FOXP3 was carried out in 15,000–25,000 CD4^+^CD25^high^ T-cells using the PE Anti-Human FOXP3 Staining Set (eBioscience) following the recommended protocol. Cells were first fixed, washed and permeablized, then stained with either FOXP3-PE (clone PCH101) or the isotype control rat IgG2a-PE. Intracellularly stained cells were then analyzed on a FACS LSRII using FACSDiva Software (BD Biosciences).

### Cytokine measurement from CD4^+^CD25^low^ cells

CD4^+^CD25^low^ cells were plated at 50,000 cells per well and stimulated with platebound αCD3 (5 µg/ml) and soluble aCD28 (2.5 µg/ml) (BD Biosciences) at 37°C/5%CO2. After 55 hours, 100 ul of supernatant was removed from each well and stored at −80°C until the cytokines were measured. The supernatants were thawed and 50 µl was used in the Cytometric Bead Array (CBA) Human Th1/Th2 Cytokine Kit (BD Biosciences). The assay was performed according to the manufacturer's recommended protocol. The samples were acquired on a FACSCalibur (BD Biosciences) following the cytometer setup protocol. The FACS data was analyzed using the CBA 6 bead analysis software (BD Biosciences).

### Apoptosis assay during cytokine deprivation

CD4^+^CD25^high^ and CD4^+^CD25^−^ T-cells were isolated from 6 buffy coats by FACS sorting as previously described in the cell isolation procedure above. Cells were plated at 75,000 cells per well under the following 6 conditions: TCR-Co (1 µg/ml platebound αCD3, clone UCHT1, Ancell and 0.5 µg/ml soluble aCD28, clone CD28.2, BD Biosciences); TCR-Co+IL-2 (200 U/ml, human-recombinant, BD Biosciences); TCR-Co+anti-IL-2 (2 µg/ml, clone MQ1-17H12, BD Biosciences); TCR-Co+IL-4 (200 U/ml, recombinant, BD Biosciences); TCR-Co+anti-IL-4 (1 µg/ml, clone MP4-25D2, BD Biosciences); and TCR-Co+anti-IL-2 (2 µg/ml)+anti-IL-4 (1 µg/ml). The cells were incubated at 37°C/5% CO_2_ and at the indicated time points (12, 24, 72 and 120 hours), the cells were removed from the wells and 25,000 cells were used to measure apoptosis as previously described in methods above. The remaining 50,000 cells were resuspended in TRIzol Reagent (Invitrogen) and stored at −80°C until RNA could be isolated.

## Supporting Information

Figure S1 Raw proliferation counts (cpm) for suppression assay. T-cell proliferation (cpm) in media only (background), single culture (only CD25- Teffs) and in co-culture (Tregs and CD25- Teffs) for both the phenotypic groups is shown for the suppression results shown in Figure 1 in the manuscript. Values shown are mean±SE across the number of samples indicated for Figure 1.
(0.03 MB TIF)

Figure S2 Representative flow cytometry plot for measurement of Treg Apoptosis. Figure shows one representative FACS plot for measurement of Treg apoptosis, as described in the methods section. Apoptosis was measured as the percentage of apoptotic cells YOPRO1+ve/7AAD-ve (green) amongst live cells (total 7AAD-ve cells comprising both YOPRO1+ve and YOPRO1-ve cells, blue+green).
(0.09 MB TIF)

Figure S3 Cytokine production from CD4+CD25- and CD4+CD25low effector T cell subsets. Production of some important cytokines was measured by a CBA assay, as described in the methods. Results are average values across indicated number of subjects, for (A) CD4+CD25- and (B) CD4+CD25low effector T-cell subsets.
(0.03 MB TIF)

Figure S4 Purity of FACS isolated CD4+CD25high T-cells. (A) Standardized sorting procedure based on gating of top 1.2% CD25-expressing CD4+ T-cells as CD4+CD25high (Tregs). Both CD4+CD25- and CD4+CD25high cell subsets were checked for CD127 expression. Most of CD4+CD25high T-cells do not express CD127 (80.5%) and most of CD4+CD25- -T-cells do express CD127 (87.1%). This is representative of 4 samples. (B) Intracellular staining for FOXP3 in CD4+CD25- and CD4+CD25high T-cells.
(0.04 MB TIF)

Figure S5 Quality assessment of the arrays using AffyQC package. These plots assess the overall signal quality for the arrays. (A) Boxplots of all the pm (perfect match) intensities for 12 T1D subjects (left) and 15 control subjects (right). (B) Density plot of the intensities (log scale) for 12 T1D subjects (left) and 15 control subjects (right). These plots suggest that arrays used in this study are good quality as none of the arrays have a low average intensity or a significantly different shaped density. (C) RNA digestion plot for (left) 15 control subjects and (right) 12 T1D subjects. The mean intensity of expression of all genes on each array is plotted as a function of 5′-3′ position of probes. For each array and within each probe-set, probes are arranged by their proximity to the 5′ end of the gene. The plot shows the average intensity of the probes as a function of 5′-3′ position of probes. Each line corresponds to an array and the slope of its trend indicates potential RNA degradation of the genetic material hybridized to the array. Parallel lines indicate similar RNA degradation patterns across arrays.
(0.14 MB TIF)

Figure S6 Flowchart of the gene expression analysis pipeline
(0.03 MB TIF)

Figure S7 BGX measures and estimation of differentially expressed genes. These plots summarise the main steps of the BGX algorithm. (A) Kernel density plots are calculated for the expression of each gene (n = 1 to 54675) in each phenotype, from the cumulative information from all subjects within a phenotype. (B) The corresponding plots of the posterior distribution of the expression differences are calculated for each gene across the two phenotypes. (C) Histogram of the posterior distribution of expression differences. Under the null hypothesis, the histogram of the posterior distribution of expression differences P(dg<0) will be unimodal with a mode of 0.5 and have smoothly decreasing tails. Towards the two tails of the histogram, the observed deviations from the expected shape indicate the presence of differentially expressed genes. The black curve is the expected distribution (by Efron's method [79]) and the grey curve is the observed distribution. Excess of P(dg<0) values near zero and one indicate over-expressed and under-expressed genes in phenotype 2 (T1D subjects) relative to phenotype 1 (Control subjects), respectively. In the final step, genes are ranked using the standardized BGX difference (z-score) between the two conditions, which takes into account the estimated difference in expression level as well as the associated uncertainty.
(0.06 MB TIF)
