# Supplementary figures and images for: Apoptosis of CD4+CD25high T Cells in Type 1 Diabetes May Be Partially Mediated by IL-2 Deprivation
Source: PLoS One. 2009 Aug 5;4(8):e6527. doi: 10.1371/journal.pone.0006527 (PMC2716541; doi:10.1371/journal.pone.0006527)

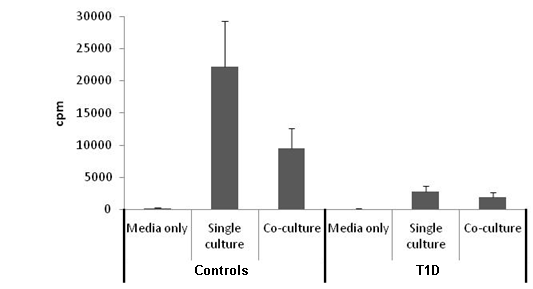

Supplement: Figure S1 — Raw proliferation counts (cpm) for suppression assay. T-cell proliferation (cpm) in media only (background), single culture (only CD25- Teffs) and in co-culture (Tregs and CD25- Teffs) for both the phenotypic groups is shown for the suppression results shown in Figure 1 in the manuscript. Values shown are mean±SE across the number of samples indicated for Figure 1. (0.03 MB TIF) [file pone.0006527.s001.tif]

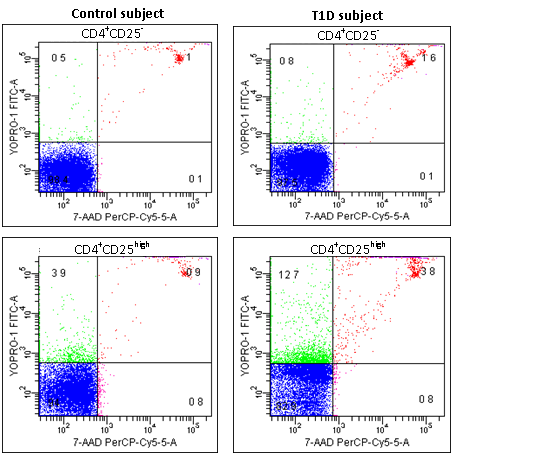

Supplement: Figure S2 — Representative flow cytometry plot for measurement of Treg Apoptosis. Figure shows one representative FACS plot for measurement of Treg apoptosis, as described in the methods section. Apoptosis was measured as the percentage of apoptotic cells YOPRO1+ve/7AAD-ve (green) amongst live cells (total 7AAD-ve cells comprising both YOPRO1+ve and YOPRO1-ve cells, blue+green). (0.09 MB TIF) [file pone.0006527.s002.tif]

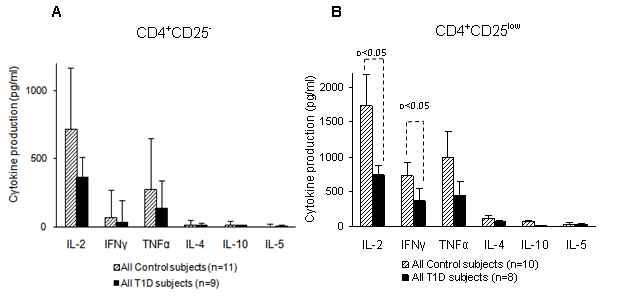

Supplement: Figure S3 — Cytokine production from CD4+CD25- and CD4+CD25low effector T cell subsets. Production of some important cytokines was measured by a CBA assay, as described in the methods. Results are average values across indicated number of subjects, for (A) CD4+CD25- and (B) CD4+CD25low effector T-cell subsets. (0.03 MB TIF) [file pone.0006527.s003.tif]

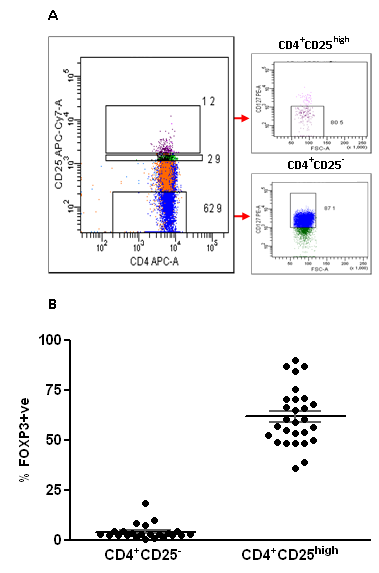

Supplement: Figure S4 — Purity of FACS isolated CD4+CD25high T-cells. (A) Standardized sorting procedure based on gating of top 1.2% CD25-expressing CD4+ T-cells as CD4+CD25high (Tregs). Both CD4+CD25- and CD4+CD25high cell subsets were checked for CD127 expression. Most of CD4+CD25high T-cells do not express CD127 (80.5%) and most of CD4+CD25- -T-cells do express CD127 (87.1%). This is representative of 4 samples. (B) Intracellular staining for FOXP3 in CD4+CD25- and CD4+CD25high T-cells. (0.04 MB TIF) [file pone.0006527.s004.tif]

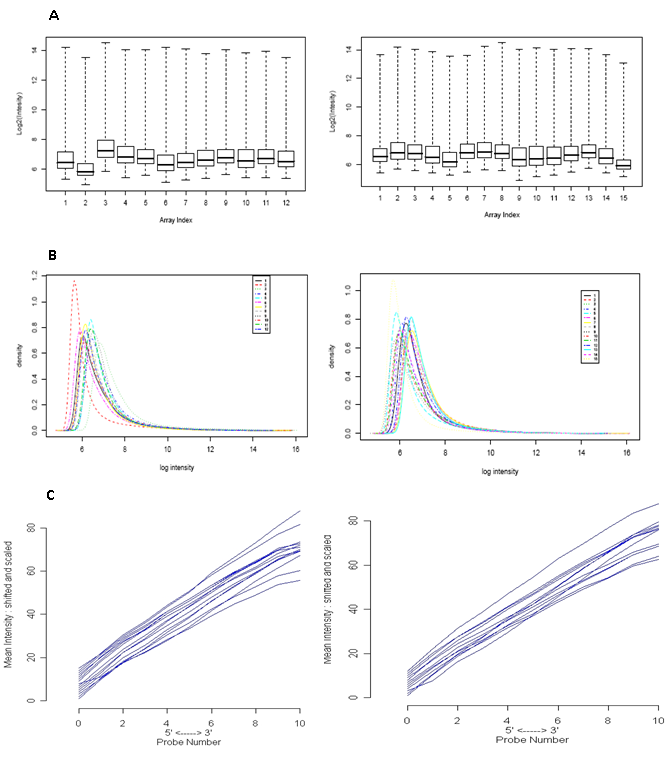

Supplement: Figure S5 — Quality assessment of the arrays using AffyQC package. These plots assess the overall signal quality for the arrays. (A) Boxplots of all the pm (perfect match) intensities for 12 T1D subjects (left) and 15 control subjects (right). (B) Density plot of the intensities (log scale) for 12 T1D subjects (left) and 15 control subjects (right). These plots suggest that arrays used in this study are good quality as none of the arrays have a low average intensity or a significantly different shaped density. (C) RNA digestion plot for (left) 15 control subjects and (right) 12 T1D subjects. The mean intensity of expression of all genes on each array is plotted as a function of 5′-3′ position of probes. For each array and within each probe-set, probes are arranged by their proximity to the 5′ end of the gene. The plot shows the average intensity of the probes as a function of 5′-3′ position of probes. Each line corresponds to an array and the slope of its trend indicates potential RNA degradation of the genetic material hybridized to the array. Parallel lines indicate similar RNA degradation patterns across arrays. (0.14 MB TIF) [file pone.0006527.s005.tif]

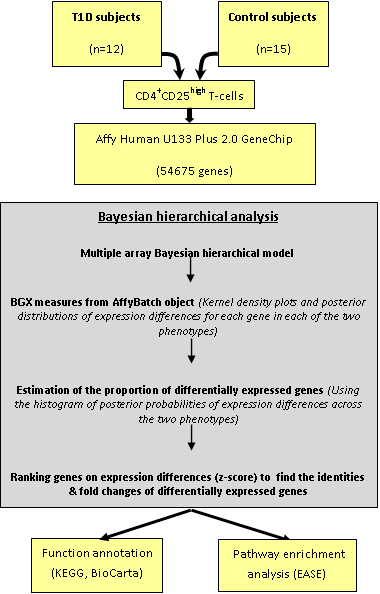

Supplement: Figure S6 — Flowchart of the gene expression analysis pipeline (0.03 MB TIF) [file pone.0006527.s006.tif]

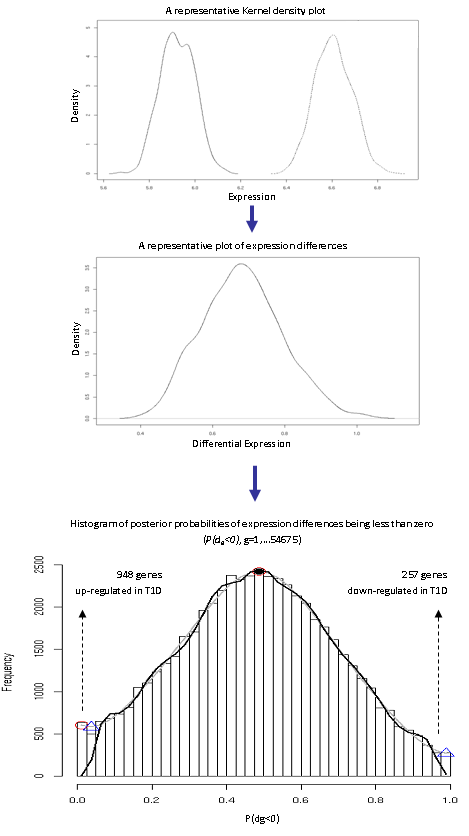

Supplement: Figure S7 — BGX measures and estimation of differentially expressed genes. These plots summarise the main steps of the BGX algorithm. (A) Kernel density plots are calculated for the expression of each gene (n = 1 to 54675) in each phenotype, from the cumulative information from all subjects within a phenotype. (B) The corresponding plots of the posterior distribution of the expression differences are calculated for each gene across the two phenotypes. (C) Histogram of the posterior distribution of expression differences. Under the null hypothesis, the histogram of the posterior distribution of expression differences P(dg<0) will be unimodal with a mode of 0.5 and have smoothly decreasing tails. Towards the two tails of the histogram, the observed deviations from the expected shape indicate the presence of differentially expressed genes. The black curve is the expected distribution (by Efron's method [79]) and the grey curve is the observed distribution. Excess of P(dg<0) values near zero and one indicate over-expressed and under-expressed genes in phenotype 2 (T1D subjects) relative to phenotype 1 (Control subjects), respectively. In the final step, genes are ranked using the standardized BGX difference (z-score) between the two conditions, which takes into account the estimated difference in expression level as well as the associated uncertainty. (0.06 MB TIF) [file pone.0006527.s007.tif]
